# DNA methylation and transcriptional noise

**DOI:** 10.1186/1756-8935-6-9

**Published:** 2013-04-26

**Authors:** Iksoo Huh, Jia Zeng, Taesung Park, Soojin V Yi

**Affiliations:** 1 Department of Statistics, Bioinformatics and Biostatistics Laboratory, Interdisciplinary Program in Bioinformatics, Seoul National University, Seoul, 151-742, Korea; 2 School of Biology, Institute of Bioengineering and Biosciences, Georgia Institute of Technology, 310 Ferst Drive, Atlanta, GA, 30332, USA

**Keywords:** DNA methylation, Gene expression, Spurious transcription, Transcriptional noise

## Abstract

**Background:**

DNA methylation is one of the most phylogenetically widespread epigenetic modifications of genomic DNA. In particular, DNA methylation of transcription units (‘gene bodies’) is highly conserved across diverse taxa. However, the functional role of gene body methylation is not yet fully understood. A long-standing hypothesis posits that gene body methylation reduces transcriptional noise associated with spurious transcription of genes. Despite the plausibility of this hypothesis, an explicit test of this hypothesis has not been performed until now.

**Results:**

Using nucleotide-resolution data on genomic DNA methylation and abundant microarray data, here we investigate the relationship between DNA methylation and transcriptional noise. Transcriptional noise measured from microarrays scales down with expression abundance, confirming findings from single-cell studies. We show that gene body methylation is significantly negatively associated with transcriptional noise when examined in the context of other biological factors.

**Conclusions:**

This finding supports the hypothesis that gene body methylation suppresses transcriptional noise. Heavy methylation of vertebrate genomes may have evolved as a global regulatory mechanism to control for transcriptional noise. In contrast, promoter methylation exhibits positive correlations with the level of transcriptional noise. We hypothesize that methylated promoters tend to undergo more frequent transcriptional bursts than those that avoid DNA methylation.

## Background

DNA methylation at CpG dinucleotides is a key epigenetic modification in the human genome crucial for regulatory and developmental processes [1,2]. The degree of DNA methylation in the human genome is extensive: most CpG dinucleotides are methylated in most tissues and developmental stages examined [3-6]. In particular, transcription units, or so-called ‘gene bodies’, are even more heavily methylated than the surrounding intergenic regions [6-9].

The functional consequences of promoter methylation on chromatin configuration and transcriptional regulation are extensively documented (see, for example, [10-12]). There is also considerable evidence suggesting that DNA methylation suppresses proliferation of transposable elements (TEs) [13-15]. However, the role of gene body methylation remains largely unresolved. Recently, studies have begun to identify molecular consequences of gene body methylation. For example, gene body methylation affects pol II occupancy and histone modifications [16]. Differential levels of DNA methylation between different exons have been linked to differential inclusion and exclusion of specific exons in transcripts [17,18]. Gene body methylation may also occur as a byproduct of transcriptional processes [19]. Another possibility is that gene body methylation is simply an extension of methylation of TEs; many genes harbor TEs within their transcription units, and the main role of methylation is to suppress the proliferation of these TEs [15].

Nevertheless, the main role of gene body DNA methylation remains unresolved. In fact, it is considered as one of the most long-standing open questions regarding genomic DNA methylation [20-25]. This question is even more pertinent in light of evolutionary patterns of DNA methylation. Comparative DNA methylation studies indicate that gene body methylation is the most conserved, ancestral form of genomic DNA methylation [7,9,23,26]. Thus, elucidating the role of gene body DNA methylation may provide significant insights into the evolutionary divergence of genomic DNA methylation across taxa [9,23,26,27].

A long-standing hypothesis posits that gene body DNA methylation suppresses spurious transcription within coding regions. By doing so, gene body methylation can effectively reduce ‘transcriptional noise’ [27,28]. This hypothesis is based upon the well-accepted idea that DNA methylation is generally repressive [29]. Pervasive DNA methylation of gene bodies, and the consequent suppression of transcriptional noise, may have served as a key facilitator enabling the evolution of complex vertebrate genomes [27]. Moreover, recent studies have begun to indicate that epigenetic mechanisms are deeply implicated in regulation of gene expression variability [30-33].

However, a detailed analysis of the relationship between transcriptional noise and DNA methylation has been lacking until now, due in large part to technical difficulties. Here, capitalizing on the recent progress in genomics and epigenomics, we investigated the impact of DNA methylation on transcriptional noise, using data from the human genome. Our analyses provide, for the first time, unequivocal evidence supporting the role of gene body methylation to reducing transcriptional noise. Furthermore, we show that promoter DNA methylation is also highly significantly associated with transcriptional noise.

## Results

### Transcriptional noise is negatively correlated with expression abundance and associate with specific functions

Levels of gene expression vary between cells even with the same genetic materials and under the same biological conditions [34-36]. Understanding the nature and mechanism of such variability, which is commonly referred to as ‘transcriptional noise’, has manifold functional consequences [37]. Recently, there have been significant improvements in experimental methods to measure transcriptional noise, as well as in the theoretical understanding of transcriptional noise. These studies indicate that transcriptional noise may occur due to transcriptional bursting of promoters, as well as spurious transcription within coding sequences [38-41].

Transcriptional noise in multicellular organisms, such as mammals, cannot be easily dissected using experimental means. However, they can be approximated using abundant expression datasets, for example utilizing normalized variation among microarray assays between replicates of populations [42,43]. For example, Yin *et al*. [42] compared the transcriptional noise measured from microarrays to those measured from single-cell experiments. The two results correspond remarkably well [42]. Similar results were seen in another study, comparing expression variation among populations to experimentally measured transcriptional noise [43]. Following these approaches, in this study we approximated transcriptional noise of human genes as the coefficient of variation of transcriptional abundance, assayed between replicates of populations of the same tissue samples under normal conditions (see Methods).

There have been significant recent technical improvements in analysis of genomic DNA methylation. In particular, researchers have begun to generate whole-genome maps of DNA methylation at the nucleotide level, via whole-genome sequencing of bisulfite-converted genomic DNA [5,44,45]. This method quantifies the methylation level of each CpG dinucleotide across the whole genome, enabling us to discern gene body methylation levels for individual genes.

In this study, we analyzed DNA methylation and transcriptional noise of the prefrontal cortex (brain) and the peripheral blood mononuclear cells (blood). We chose these two tissues for the following reasons. First, we decided to analyze ‘normal’ tissues (as opposed to cell lines). While there exists vast information on transcriptional variation of cell lines, gene expression profiles of cell lines are known to have significantly diverged from those of normal tissues [46]. Consequently, we chose not to consider cell lines in the current study. Second, we chose tissues whose genome-wide methylation maps are currently available. Finally, large numbers of microarray data in the ‘control’ (as opposed to disease) conditions exist for these tissues, thereby enabling us to measure transcriptional noise with confidence. We used rigorous quality control processes to curate microarray data from these tissues (see Methods). The resulting data are from the same technical platforms, and exhibit high correlation levels among experiments (Additional files 1 and 2).

**Figure 1:**
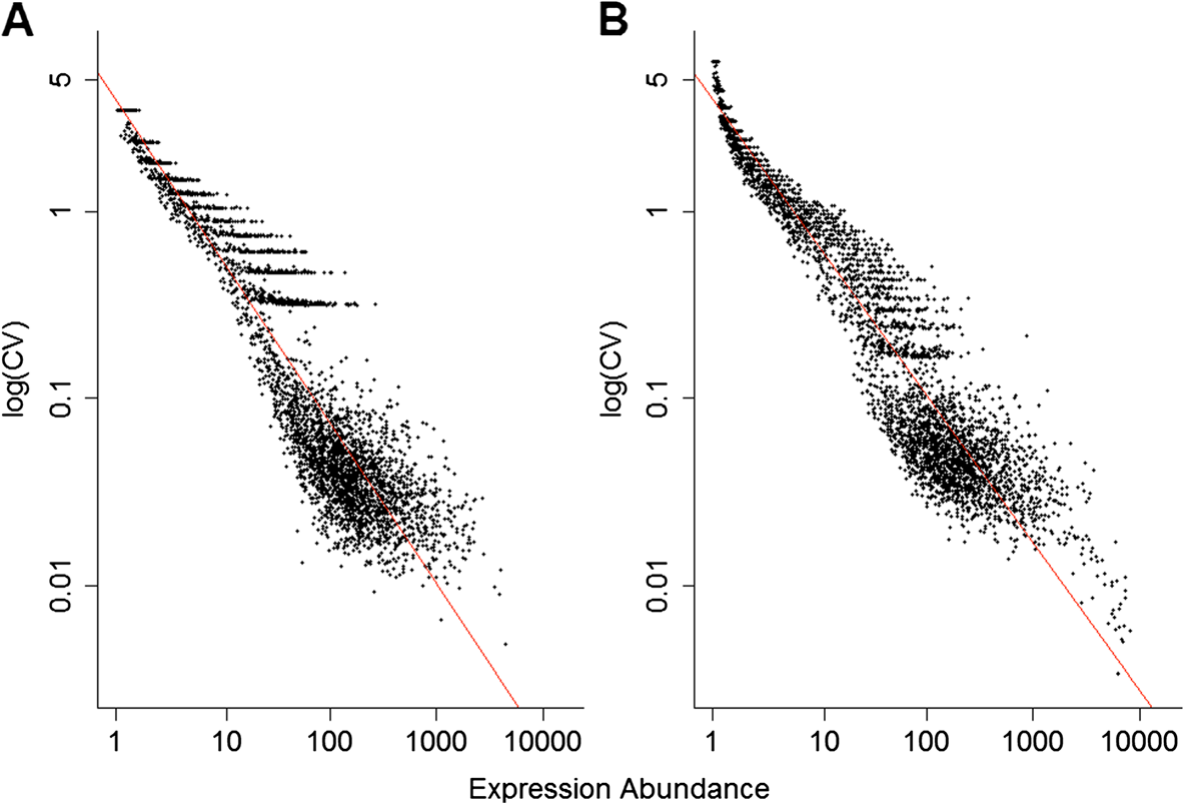
**Transcriptional noise and expression abundance are significantly negatively correlated in (A) brain, and (B) blood.** Transcriptional noise is measured as the coefficient of variation of transcriptional abundance (see Methods section). The regression coefficients between these variables are −0.60 (*P* <0.001) and −0.55 (*P* <0.001) for brain and blood, respectively.

We examined whether the transcriptional noise calculated from these curated data exhibited similar properties to those identified from previous studies. For example, from studies of yeast, genes involved in protein synthesis exhibited lower noise compared to other genes [39,40]. At the same time, genes responding to environmental signals or stress genes showed particularly high levels of noise [39,40]. We found similar patterns in the transcriptional noise of human genes (Additional file 3). One of the most striking findings from previous studies was that transcriptional noise is approximately proportional to the expression abundance [39,40]. We observed the same scaling behavior in which the transcriptional noise was negatively associated with expression abundance in both human tissues studied (Figure 1). This observation indicates that the scaling of transcriptional noise to expression abundance is likely to be a common phenomenon across diverse taxa, underscoring common molecular mechanisms, such as random birth and death processes of mRNAs [39,40,47]. It has been also proposed that transcriptional noise is minimized for essential genes [48]. However, in our data, we did not observe enrichment of low-noise genes in essential genes (Additional file 4).

### Gene body methylation and promoter methylation exhibit negative and positive associations with transcriptional noise

Our interest was in determining whether DNA methylation influences transcriptional noise. To do so, we needed to first account for the effect of expression abundance on both of these variables. This is because DNA methylation is intimately related to expression abundance [6,10,23,25], and gene expression abundance is correlated with transcriptional noise (Figure 1). In addition, other genomic variables, such as gene lengths, are also correlated with expression abundance [49,50].

Our goal was to explain the variation found in the levels of transcriptional noise using several explanatory (independent) variables. We used the following variables as explanatory variables: expression abundance, gene body methylation, promoter methylation, and gene lengths. We first examined the variance inflation factors (VIFs), which are indicators of multicolinearity among variables. None of the explanatory variables exhibited VIFs greater than 5. This demonstrated that we could assess individual contributions of each genomic trait without the influence of multicolinearity [51].

We found that, in both tissues, gene body methylation shows significant negative relations to transcriptional noise (Table 1). This is in accord with the hypothesis that gene body DNA methylation suppresses transcriptional noise [27]. As gene length increases, there may be more opportunities for spurious transcription. In other words, gene length may be positively correlated with transcriptional noise. According to our multiple linear regression analysis, however, the effect of gene length on transcriptional noise, while controlling for other factors, was negligible in the brain data, but significantly negative in the blood data (Table 1). Analyzing more tissue samples would clarify the effect of gene length on transcriptional noise. Interestingly, promoter methylation again exhibited strong positive relations with the transcriptional noise in a multiple linear regression setting (Table 1).

**Table 1:** Multiple linear regression models explaining variation of transcriptional noise in different tissues

**Predictors**	**Estimate of β**	**t value**	**Significance**	**VIF**
Brain				
Intercept	1.47	19.51	<10^-4^	
Expression abundance	−0.59	−180.50	<10^-4^	1.21
Gene body methylation^a^	−0.28	−4.74	<10^-4^	1.96
Promoter methylation	0.20	4.94	<10^-4^	1.27
Log (gene length)^a^	0.00092	0.099	0.921	2.19
Adjusted R^2^				0.87
Blood				
Intercept	1.89	28.92	<10^-4^	
Expression abundance	−0.55	−237.24	<10^-4^	1.11
Gene body methylation^a^	−0.37	−6.68	<10^-4^	1.27
Promoter methylation	0.29	7.36	<10^-4^	1.65
Log (gene length)^1^	−0.038	−5.09	<10^-4^	1.79
Adjusted R^2^				0.92

^a^Exclusive of transposable elements.

VIF, variance inflation factor.

In the above analyses, we analyzed gene body methylation levels after removing TEs. We also sought to include methylation of TEs specifically in our model, using the following method. We first estimated methylation levels of gene bodies that are identified as TEs according to RepeatMasker [52]. Then we included this methylation level of TEs found within each gene as a separate variable in a multiple linear regression setting. The length of TEs themselves within each gene could not be included in this model because they exhibited high VIFs (7.39 in brain, 6.62 in blood, respectively), and thus could cause multicolinearity problems. The results of this analysis, presented in Table 2, demonstrate that TE methylation is significantly negatively correlated with transcriptional noise. In other words, TE methylation may also contribute to reducing transcriptional noise. The regression coefficients of other variables are highly similar to those from Table 1, indicating that the effects of other variables are not highly influenced by the level of TE methylation.

**Table 2:** Multiple linear regression models explaining variation of transcriptional noise in different tissues

**Predictors**	**Estimate of β**	**t value**	**Significance**	**VIF**
Brain				
Intercept	1.47	19.57	<0.0001	
Expression abundance	−0.59	−180.78	<0.0001	1.12
Gene body methylation^1^	−0.19	−3.16	0.0016	2.34
TE methylation	−0.23	−5.78	<0.0001	1.44
Promoter methylation	0.18	4.54	<0.0001	1.28
Log (gene length)^a^	0.015	1.54	0.12	2.10
Adjusted R^2^				0.87
Blood				
Intercept	1.87	28.65	<0.0001	
Expression abundance	−0.55	−236.94	<0.0001	1.11
Gene body methylation^1^	−0.28	−4.95	<0.0001	1.93
TE methylation	−0.22	−5.77	<0.0001	1.43
Promoter methylation	0.27	6.88	<0.0001	1.28
Log (gene length)^a^	−0.025	−3.19	0.0014	1.81
Adjusted R^2^				0.92

^a^Exclusive of transposable elements.

TE, transposable element; VIF, variance inflation factor.

To attest that our results were not biased due to statistical outliers, we next performed robust regression analyses using the same explanatory variables. We used several available methods including quantile regression as well as a few well-known loss functions such as bisquare, and Hampel and Huber ([53-55]; see also Methods section). The results of these analyses (Table 3 and Additional file 5) were unanimously consistent with the previous results, indicating highly significant negative associations between the level of gene body DNA methylation and transcriptional noise, and highly significant positive associations between promoter DNA methylation and transcriptional noise. In conclusion, these analyses reveal that after controlling for other factors, gene body methylation and promoter methylation are negatively and positively correlated with transcriptional noise, respectively.

**Table 3:** **Robust regression analyses (quantile regression for median) for the model used in Table**1

**Predictors**	**Estimate of β**	**t value**	**Significance**
Brain			
Intercept	1.53	19.51	<0.0001
Expression abundance	−0.61	−188.65	<0.0001
Gene body methylation^a^	−0.26	−4.30	<0.0001
Promoter methylation	0.13	3.76	0.0002
Log (gene length)^a^	0.0008	0.0885	0.3762
Blood			
Intercept	1.82	28.75	<0.0001
Expression abundance	−0.55	−237.24	<0.0001
Gene body methylation^a^	−0.28	−4.65	<0.0001
Promoter methylation	0.20	5.38	<0.0001
Log (gene length)^a^	−0.03	−5.09	<0.0001

^a^Exclusive of transposable elements.

### Accounting for technical noise and among individual variability of DNA methylation

One potential caveat of our approach is the presence of technical noise, or variation of gene expression caused by technical variation among experiments, on the level of gene expression variability. Our interest is in the biological variability of gene expression. As defined previously, we approximated ‘transcriptional noise’ as the coefficient of variation (CV) among the replicates of expression data, as used previously [42]. However, this measure of gene expression variability is a composite of biological noise, which is our main interest, plus technical variation among experiments. This is problematic because it is possible that technical noise might be confounded with biological noise. For example, technical variation among experiments is negatively correlated with the expression level of genes [56,57]. Thus, it is important to take into account the impact of technical noise in assessing the relationship between biological noise and DNA methylation.

To address this issue, we used a dataset on technical and biological replicates of blood gene expression. In this dataset, gene expression is measured in two sets of technical replicates across two biological experiments [58]. Using this data, we can decompose total variation of gene expression into ‘biological’ versus ‘technical’ variation. Specifically, for a specific gene using *y*_*ij*_ as the expression level of the *j*th technical replicate from the *i*th biological sample, decomposition of variance can be expressed as in Equation 1 below:

(1) $$\sum_{i=1}^{2}\sum_{j=1}^{2}{\left(y_{\mathrm{ij}}-\bar{\bar{y}}\right)}^{2}\;=2\sum_{i=1}^{2}{\left({\bar{y}}_{i}-\bar{\bar{y}}\right)}^{2}+\sum_{i=1}^{2}\sum_{j=1}^{2}{\left(y_{\mathrm{ij}}-\bar{\bar{y}}\right)}^{2}$$

The left term represents the total sum of square in a gene; the first term on the right-hand side is the biological sum of squares and the second term is the technical sum of squares. Using this decomposition, we can then assess the effect of gene body methylation on the pure biological variation and on the technical variation, separately. In our first analysis, we used the biological sum of squares as the response variable, and examined the statistical effects of several predictor variables. The results of this analysis showed that gene body methylation has a significant negative effect on biological variability among samples (model 1 in Table 4). In the second analysis, we used the total sum of square as the response variable and the technical sum of square as an explanatory variable. The results from this analysis again indicated that the effect of gene body methylation on ‘biological’ transcriptional noise, after adjusting for the technical noise, is negative (model 2 in Table 4). Thus, both methods provide consistent support to our finding that gene body methylation is negatively correlated with biological variation of gene expression.

**Table 4:** Multiple linear regression models in which technical versus biological components of transcriptional noise are separately analyzed

**Predictors**	**Estimate of β**	**t value**	**Significance**	**VIF**
Model 1^a^				
Intercept	1.201	14.12	<10^-4^	
Expression	−0.442	−78.19	<10^-4^	1.06
Gene body methylation	−0.797	−7.33	<10^-4^	1.07
Promoter methylation	0.613	6.17	<10^-4^	1.06
Adjusted R^2^				0.53
Model 2^b^				
Intercept	0.769	11.157	<10^-4^	
Expression	−0.337	−61.354	<10^-4^	3.39
Gene body methylation	−0.566	−9.463	<10^-4^	1.10
Promoter methylation	0.431	7.969	<10^-4^	1.07
Technical noise	0.608	32.467	<10^-4^	3.30
Adjusted R^2^				0.82

^a^Model 1 used CV calculated from biological component as response variable.

^b^Model 2 used CV calculated from total variation as response variable.

CV, coefficient of variation; VIF, variance inflation factor.

Another source of variability that needs to be accounted for is variation of DNA methylation between individuals. To determine the influence of between-individual variability of DNA methylation on our results, we analyzed datasets on gene body DNA methylation from the brains of three individuals [6]. We constructed an augmented regression model, allowing the effect of gene body methylation to vary across individuals. We defined an index for each individual as an ‘individual factor’ and included it in the new model. In addition, we included interaction terms between individual factors and gene body methylation to this model. The results of these analyses (Table 5, and Additional file 6) indicate that between-individual variations of gene body methylation do not affect our findings.

**Table 5:** Regression analysis accounting for individual variation indicates little effect of between-individual variability of DNA methylation on transcriptional noise

**Predictors**	**Sum of square**	**Degrees of freedom (df)**	**F value**	***P *value**	**VIF**^**a**^
Intercept	214.6	1	578.390	<10^-4^	1.03
Expression	28,164.2	1	75,900.35	<10^-4^	1.30
Gene length	7.6	1	20.352	<10^-4^	2.00
Gene body methylation	17.3	1	46.541	<10^-4^	1.04
Promoter methylation	43.2	1	116.420	<10^-4^	3.89
Individual	0.3	2	0.430	0.651	4.05
Individual:gene body methylation	0.3	2	0.455	0.634	
Adjusted R^2^				0.87	

^a^Variation inflation factor (VIF) approximated as (generalized VIF)^1/(2*df)^[59].

## Discussion

The human genome and other vertebrate genomes are heavily methylated in most tissues and developmental stages, a pattern referred to as ‘global’ DNA methylation [23]. This pattern is very different from what is observed in other animals and plants. In most invertebrates examined, DNA methylation is targeted to the transcription units (gene bodies) of a subset of genes [7,9,23]. Notably, gene body methylation appears to have existed well before the emergence of DNA methylation of promoters and TEs, as an ancestral form of DNA methylation in diverse animal and plant genomes [23,60,61].

Determining the role of gene body methylation is of much interest, and studies are revealing associations between gene body methylation and gene expression [9,21,62,63], transcript composition [17,18,64] and chromatin structures [16]. Nonetheless, the global role of gene body methylation remains unresolved. In this respect, two long-standing hypotheses stand out. The first hypothesis posits that gene body methylation reduces transcriptional noise [27]. Another hypothesis focuses on the impact of DNA methylation to suppress the proliferation of TEs [15]. Many TEs are found in gene bodies, thus methylation of TEs may have caused expansive methylation of gene bodies [15].

In this study we examined the predictions of these two hypotheses using whole genome methylation data and statistical methods. Because gene body methylation and transcriptional noise are both significantly correlated with expression abundance, it is important to analyze the impact of gene body methylation while considering the effect of expression abundance. We used several statistical methods to achieve this goal. We also examined the impact of noise due to technical variation among experiments, as well as between-individual variation of DNA methylation on our results. These analyses all indicate that gene body methylation, when viewed in the context of other biological factors, has a negative relationship with transcriptional noise.

Transcriptional noise is abundantly present in diverse taxa. The origin of transcriptional noise may be related to ‘transcriptional bursts’, referring to the phenomenon that transcription tends to occur in bursts [65-67]. Transcriptional noise also occurs due to transcription of non-canonical promoters within gene bodies, potentially due to the overabundance of RNA polymerase II in cellular environment [38]. Our results showing that more heavily methylated gene bodies exhibit less transcriptional noise are consistent with the idea that transcriptional noise is reduced by pervasive gene body methylation. Alternatively, the negative relationship between gene body DNA methylation and transcriptional noise may reflect an indirect association due to a third, yet unknown biological factor(s) that influence both variables.

The details of the actual underlying molecular mechanisms of such process are yet to be fully characterized. There are some well established epigenetic modifications of gene bodies are shown to directly suppress the initiation of non-canonical transcripts within coding sequences [68-70]. Emerging evidence indicate that gene body DNA methylation is likely to complement or function together with other epigenetic modifications to generate chromatin states that are repressive of the initiation and elongation of spurious transcripts. For example, DNA methylation of gene bodies reduces the efficiency of transcriptional elongation, by excluding RNA polymerase II occupancy and recruiting several repressive histone marks [16]. Gene body DNA methylation effectively excludes deposition of the histone variant H2A.Z, which tend to mark lowly expressed genes with high expression variability among tissues and biological conditions [71]. The identities of molecular components of the crosstalk between DNA methylation and histone modifications continue to be discovered (see, for example, [72]).

Interestingly, our analyses indicate that promoter DNA methylation is positively correlated with the level of transcriptional noise. The underlying molecular mechanism of this phenomenon is of great interest. One possibility is that this is related to the intrinsic susceptibility of specific promoters toward transcriptional bursting. In the simplest case, promoters appear to switch randomly between ‘ON’ and ‘OFF’ states with respect to the initiation of transcription [37,47]. Some promoters, however, remain perpetually in the ‘ON’ state (permissive to transcription) and do not exhibit bursting [73]. Such promoters exhibit less transcriptional variability compared to those undergoing switches between different transcriptional states [73]. In other words, the degree of transcriptional bursting likely varies between promoters according to their propensity toward different transcriptional states, leading to different levels of transcriptional noise among genes.

Given that there exists considerable evidence that unmethylated promoters can maintain a ‘permissive’ chromatin state [72,74], we hypothesize the following: promoters with lower level of DNA methylation are more likely to adopt and maintain a permissive transcriptional state (similar to the ‘ON’ state referred to above) and exhibit little transcriptional bursting. However, promoters that are more susceptible to DNA methylation may be more likely to undergo stochastic fluctuations between different states, facilitating transcriptional bursts, and as a consequence exhibit increased transcriptional noise. The actual molecular mechanisms underlying these processes are again likely to involve highly orchestrated interactions between DNA methylation and other epigenetic mechanisms: in particular, studies in yeast have revealed the role of nucleosome positioning in regulation of gene expression variability [31,33].

Reducing transcriptional noise is particularly important for genes that perform housekeeping functions and are therefore constantly expressed [28]. Indeed, methylation maps of distantly related animal genomes reveal that gene body methylation usually targets genes that function in ‘housekeeping’ cellular processes [26,28,75]. Thus, we hypothesize that gene body methylation functions as a primary mechanism to suppress transcriptional noise of essential housekeeping genes in diverse organisms. Gene body DNA methylation is the main mode of DNA methylation in many invertebrate species. Reducing transcriptional noise may serve as the primary function of DNA methylation in such genomes. Furthermore, the human genome is characterized by heavy transcription of non-coding regions [76,77]. Global methylation of the whole genome may have evolved as a molecular mechanism to reduce global transcriptional noise [27].

Moreover, we found that methylation of TEs within gene bodies also contributes to the suppression of transcriptional noise. Several studies now indicate that methylation of TEs may have evolved after the evolution of gene body methylation [23,61]. It will be interesting to determine whether the origin of TE methylation is related to its function to reduce intragenic transcriptional noise. Our study cannot provide a clear resolution to this question. Analyses of genomic methylation patterns of species straddling the invertebrate-vertebrate boundaries (near the origin of global DNA methylation) will be informative to determine the evolutionary sequences of these processes.

**Figure 2:**
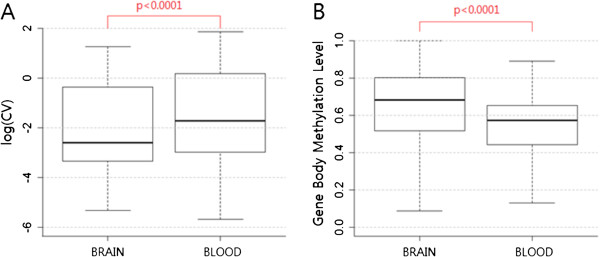
**Comparison of gene expression noise and DNA methylation between studied tissues (A) Comparison of mean transcriptional noise between the two tissues.** The brain exhibits significantly lower transcriptional noise compared to blood (paired t test, *P* <0.001). (**B**) Methylation levels, however, are significantly higher in the brain compared to blood (paired t test, *P* <0.001). We only used genes for which methylation and transcriptional noise data exist in both tissues (total no. of genes = 3,644).

DNA methylation is known to vary among different tissues [5,6]. Given the potential role of gene body methylation in regulating transcriptional noise, it is possible that among-tissue variation of DNA methylation levels may be related to among-tissue variation of transcriptional noise. In our data, the prefrontal cortex (brain) exhibited higher methylation levels than blood (*P* <0.0001 by Mann–Whitney test, Figure 2). Since gene body DNA methylation is negatively correlated with transcriptional noise, we tested whether the brain exhibits lower noise compared to blood. Indeed, we found that prefrontal cortex samples (brain) exhibited significantly lower transcriptional noise compared to blood samples (Figure 2). Thus, regulation of transcriptional noise may be one mechanism determining tissue-specific or cell type-specific levels of gene body DNA methylation.

## Conclusion

We explored the relationship between transcriptional noise and DNA methylation, using gene expression variability among different populations of cells as a proxy for transcriptional noise. Our analysis confirms the inverse relationship between gene expression abundance and transcriptional noise, while revealing novel relationships between DNA methylation and transcriptional noise. In particular gene body DNA methylation exhibits a negative correlation with transcriptional noise. This observation supports a longstanding hypothesis that gene body DNA methylation may reduce transcriptional noise. In light of evolutionary findings that gene body methylation is a widespread, conserved form of DNA methylation, the ancestral role of DNA methylation may have been related to the reduction of transcriptional noise. On the other hand, promoter DNA methylation is positively related to transcriptional noise, raising the possibility that epigenetic status of promoters may affect transcriptional bursts.

## Methods

### Data sources

Gene expression data was obtained from National Center for Biotechnology Information (NCBI) Gene Expression Omnibus (http://www.ncbi.nlm.nih.gov/geo/) (Additional file 7). Because there are considerable technical variations between platforms, we restricted platforms to only the Affymetrix Human Genome U133 series. After quality control, we obtained a total of 52 datasets (12 datasets for the prefrontal cortex and 40 datasets for blood). Gene lengths were determined based upon the RefSeq annotation provided by the UCSC genome browser. Nucleotide resolution whole DNA methylation maps of the human prefrontal cortex (brain) were obtained from a recent study ([6], data available at NCBI Gene Omnibus under the record number GSE37202). DNA methylation maps of mature peripheral blood mononuclear cells were from Li *et al*. [44], generated using a similar method.

### DNA methylation

To obtain gene body methylation levels of non-repetitive portions of genes, we used the annotation of TEs from the RepeatMasker database (http://www.repeatmasker.org). A custom Perl script was used to mask the TEs in gene bodies. For each mapped cytosine, the fractional methylation value was calculated as: total number of ‘C’ reads/(total number of ‘C’ reads + total number of ‘T’ reads), following previous studies [5,8,44]. We then calculated the fractional methylation level of each transcription unit, using the RefSeq database of hg18. Gene body methylation level for each gene was estimated as the mean fractional methylation value for all the mapped cytosines within each transcription unit. When alternative transcripts were present, we chose the longest transcript for each gene. The promoter methylation level for each gene was estimated as fractional methylation for regions spanning 1,500 bp upstream and 500 bp downstream of the transcription start site (TSS), similar to Zeng *et al*. [6].

### Microarray data processing

Microarray raw data files were first processed using raw intensity using the MAS5.0 method [78]. Using other normalization methods provided similar results. We used the median probe intensities assigned to each gene as gene expression levels. We then analyzed correlation between pairwise samples, to assess similarities between datasets from the same tissue. Datasets within the same tissues exhibiting correlation coefficient greater than 0.8 are included in this study (Additional files 1 and 2). Quantile normalization using the R package ‘preprocesscore’ [79] was conducted within each tissue. Transcriptional noise was defined as the coefficient of variation (CV: standard deviation/mean) of transcriptional abundance within each tissue, following Yin *et al*. [42].

### Multiple linear regression models of transcriptional noise

We performed multiple linear regression analyses to elucidate relationships between transcriptional noise and several biological factors (gene expression abundance, gene body methylation, promoter methylation, and gene lengths) simultaneously. CV and gene length were log transformed to improve normality. Our analyses indicated that the gender is not a significant variable and thus excluded from further analyses. We also examined the significance of the interaction terms between predictors. The results showed that the interaction terms were generally not significant and they were therefore removed from subsequent analyses.

Robust regression analysis was performed using various loss functions. We summarized the result of quantile regression in Table 3. We also used other well-known loss functions such as bisquare, Hampel and Huber (Additional file 6) [53-55]. All these approaches provided consistent results to those of the ordinary least squares method. Therefore, we conclude that the significance and magnitude of the explanatory variable effect is essential.

### Functional enrichment analyses

Functional enrichment pattern of specific subsets of genes was assessed using the DAVID tools (http://david.abcc.ncifcrf.gov/) [80]. We used the list of genes included in our analyses as the background, and tested enrichments of specific gene ontology terms using the GO FAT annotation. We examined the mean transcriptional noise of genes in the two tissues and investigated the specific gene ontology terms for top 5% high transcriptional noise genes and 5% low transcriptional noise genes. A Benjamini multiple testing correction of the EASE score (a modified Fisher exact *P* value) was used to determine statistical significance of gene enrichment.

## Competing interests

The authors declare that they have no competing interests.

## Authors’ contributions

TP and SVY designed the experiments. IH performed most of the statistical analyses. JZ and SVY generated and analyzed methylation data. TP and SVY coadvised the analyses. All authors read and approved the final manuscript.

## Supplementary Material

Additional file 1 Correlation between 12 brain microarray datasets used. Click here for file

Additional file 2 **Correlation between blood microarray datasets used.** For the interest of space, we only show 12 microarray datasets. The remaining data exhibit similarly high correspondence between datasets. Click here for file

Additional file 3 GO enrichment analyses of genes exhibiting high or low transcriptional noise. Click here for file

Additional file 4 No enrichment of low noise genes according to gene essentiality. Click here for file

Additional file 5 Robust regression analysis using transcriptional noise as a response variable and other biological variables as explanatory variables. Click here for file

Additional file 6 Multiple linear regression analyses incorporating individual factors indicate little effect of between-individual DNA methylation on transcriptional noise. Click here for file

Additional file 7 List of microarray datasets used in this study. Click here for file
