# Correction: DNA methylation and transcriptional noise

**DOI:** 10.1186/1756-8935-7-13

**Published:** 2014-06-30

**Authors:** Iksoo Huh, Jia Zeng, Taesung Park, Soojin V Yi

**Affiliations:** 1 Department of Statistics, Bioinformatics and Biostatistics Laboratory, Interdisciplinary Program in Bioinformatics, Seoul National University, Seoul 151-742, South Korea; 2 School of Biology, Institute of Bioengineering and Biosciences, Georgia Institute of Technology, 310 Ferst Drive, Atlanta, GA 30332, USA

## Correction

After the publication of this work [1] it was brought to the authors’ attention that there was an error in the equation (1). The last ‘y’ term in the equation should have one bar on top instead of two bars. Please see below for the correctly displayed equation.

(1) $${\sum_{i=1}^{2}\sum_{j=1}^{2}\left(y_{\mathrm{ij}}-\bar{\bar{y}}\right)}^{2}=2\times {\sum_{i=1}^{2}\left({\bar{y}}_{i}-\bar{\bar{y}}\right)}^{2}+{\sum_{i=1}^{2}\sum_{j=1}^{2}\left(y_{\mathrm{ij}}-{\bar{y}}_{i}\right)}^{2}$$

We regret any inconvenience that this inaccuracy may have caused.
