# Supplementary figures and images for: DNA methylation and transcriptional noise
Source: Epigenetics Chromatin. 2013 Apr 26;6:9. doi: 10.1186/1756-8935-6-9 (PMC3641963; doi:10.1186/1756-8935-6-9)

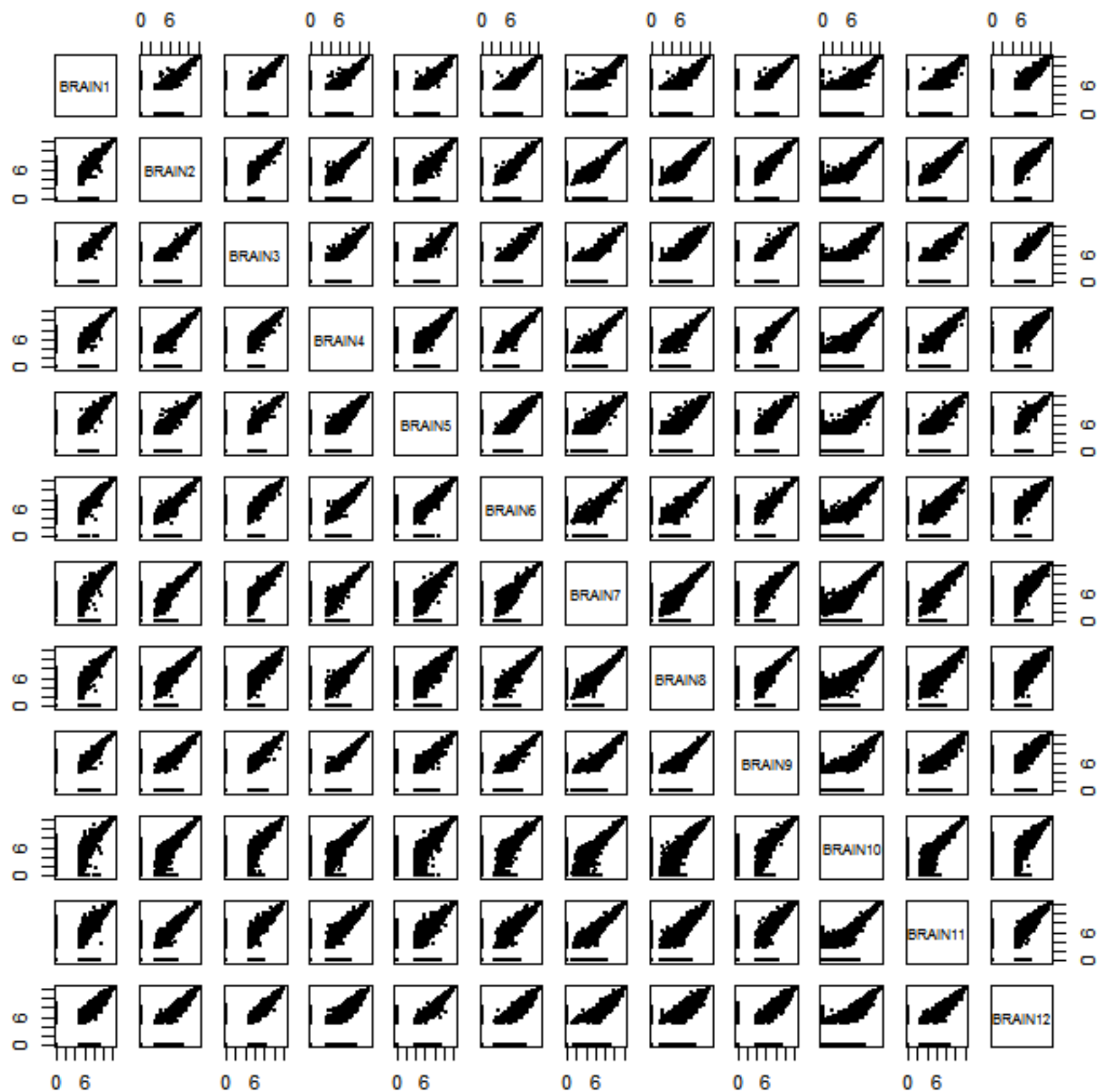

Supplement: Additional file 1 — Correlation between 12 brain microarray datasets used. [file 1756-8935-6-9-S1.pdf]

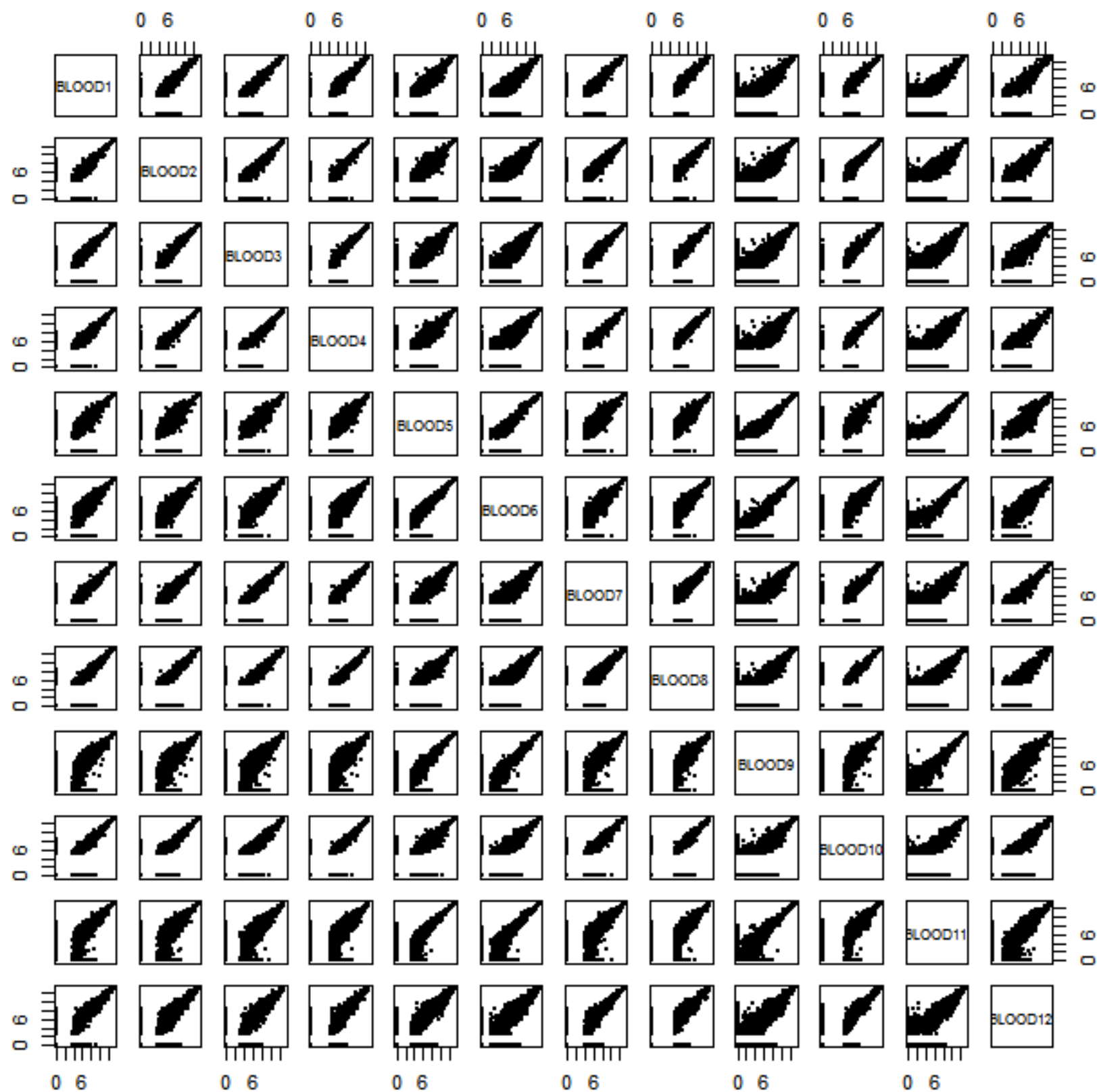

Supplement: Additional file 2 — Correlation between blood microarray datasets used. For the interest of space, we only show 12 microarray datasets. The remaining data exhibit similarly high correspondence between datasets. [file 1756-8935-6-9-S2.pdf]
