# Supplementary material for: DNA methylation and transcriptional noise
Source: Epigenetics Chromatin. 2013 Apr 26;6:9. doi: 10.1186/1756-8935-6-9 (PMC3641963; doi:10.1186/1756-8935-6-9)
Supplement: Additional file 3 — GO enrichment analyses of genes exhibiting high or low transcriptional noise. [file 1756-8935-6-9-S3.doc]

**Additional File 3.** Functional enrichments of genes with low and high transcriptional noise.

| **GO Term** | |  | **Fold Enrichment** | **P-value (Benjamini)** |
| --- | --- | --- | --- | --- |
| **Top 5% Low Transcriptional Noise Genes** | | | | |
| *Biological Function* | | |  |  |
| GO:0006414 | translational elongation | | 17.01 | 4.06 ×10^-27^ |
| GO:0006412 | translation | | 7.01 | 9.69×10^-20^ |
| GO:0042274 | ribosomal small subunit biogenesis | | 16.74 | 0.0023 |
| GO:0006091 | generation of precursor metabolites and energy | | 3.45 | 0.0026 |
| GO:0006119 | oxidative phosphorylation | | 5.02 | 0.0589 |
|  | | |  |  |
| *Cellular Components (top 5 most significant terms)* | | |  |  |
| GO:0022626 | cytosolic ribosome | | 16.34 | 3.14×10^-23^ |
| GO:0033279 | ribosomal subunit | | 11.35 | 1.51×10^-17^ |
| GO:0044445 | cytosolic part | | 10.15 | 4.47×10^-17^ |
| GO:0005840 | ribosome | | 7.73 | 7.50×10^-16^ |
| GO:0022627 | cytosolic small ribosomal subunit | | 17.76 | 2.38×10^-14^ |
|  | | |  |  |
| *Molecular Function (top 5 most significant terms)* | | |  |  |
| GO:0003735 | structural constituent of ribosome | | 11.00 | 4.14×10^-19^ |
| GO:0005198 | structural molecule activity | | 5.15 | 2.48×10^-13^ |
| GO:0003723 | RNA binding | | 3.06 | 8.31×10^-8^ |
| GO:0015078 | hydrogen ion transmembrane transporter activity | | 6.51 | 1.62×10^-5^ |
| GO:0015077 | monovalent inorganic cation transmembrane transporter activity | | 6.04 | 3.29×10^-5^ |
|  | | |  |  |
| **Top 5% High Transcriptional Noise Genes** | | | | |
| *Cellular Components* | | |  |  |
| GO:0005887 | Integral to plasma membrane | | 2.28 | 0.004 |
| GO:0031012 | extracellular matrix | | 3.79 | 0.004 |
| GO:0031226 | Intrinsic to plasma membrane | | 2.23 | 0.004 |
| GO:0005578 | Proteinaceous extracellular matrix | | 4.08 | 0.005 |
| GO:0044421 | Extracellular region part | | 2.53 | 0.006 |
|  |  | |  |  |
| *Molecular Function* | | |  |  |
| GO:0043169 | cation binding | | 1.49 | 0.0244 |
| GO:0005509 | calcium ion binding | | 2.35 | 0.0294 |
| GO:0046872 | metal ion binding | | 1.45 | 0.0337 |
| GO:0043167 | ion binding | | 1.49 | 0.0398 |
|  |  | |  |  |
