# Supplementary material for: DNA methylation and transcriptional noise
Source: Epigenetics Chromatin. 2013 Apr 26;6:9. doi: 10.1186/1756-8935-6-9 (PMC3641963; doi:10.1186/1756-8935-6-9)
Supplement: Additional file 4 — No enrichment of low noise genes according to gene essentiality. [file 1756-8935-6-9-S4.doc]

**Additional File 4. Essential Genes and Transcriptional noise**

We tested whether essential genes are enriched in low noise genes. For this purpose, we used the list of human essential genes, generated from RNAi screening (Silva et al. 2008). The data are available to download from the "online gene essentiality database" ([http://ogeedb.embl.de/#browse](http://ogeedb.embl.de/#browse)).

We divided genes into 5 bins according to their noise level. We then investigated how many of genes within each bin composed of essential versus non-essential genes. There is no enrichment of essential genes in the low noise category, determined by Fisher’s exact test (*P* > 0.05 in all bins).


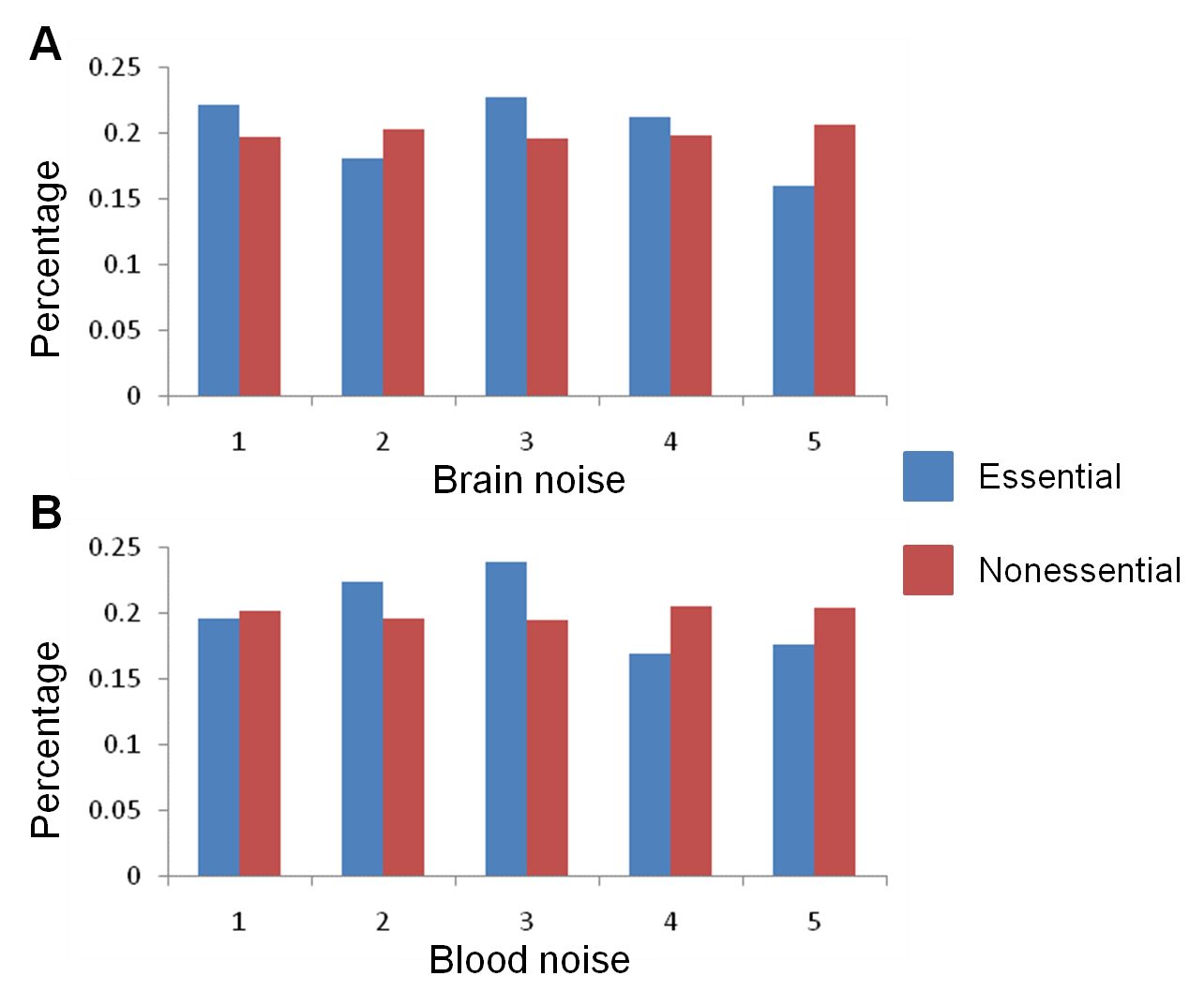


Figure S3: **No enrichment of low noise genes according to gene essentiality.** All genes are classified into 5 bins according to the noise level either in brain (A) or blood (B). The X-axis represents increasing levels of gene expression noise from left to right. The proportion of essential and non-essential genes are represented by the bar plots for each bin.

Silva, J.M., Marran, K., Parker, J.S., Silva, J., Golding, M., Schlabach, M.R., Elledge, S.J., Hannon, G.J., Chang, K., 2008. Profiling Essential Genes in Human Mammary Cells by Multiplex RNAi Screening. Science 319, 617-620.
