# Supplementary material for: DNA methylation and transcriptional noise
Source: Epigenetics Chromatin. 2013 Apr 26;6:9. doi: 10.1186/1756-8935-6-9 (PMC3641963; doi:10.1186/1756-8935-6-9)
Supplement: Additional file 5 — Robust regression analysis using transcriptional noise as a response variable and other biological variables as explanatory variables. [file 1756-8935-6-9-S5.doc]

**Additional File 5.** Results of **s**everal robust regression analyses for the model used in Table 1.

| **Predictors** | **Estimate of *β*** | **t-value** | **Significance** |
| --- | --- | --- | --- |
| Brain |  |  |  |
| Intercept |  |  |  |
| Huber | 1.51 | 19.51 | <0.0001 |
| bisquare | 1.52 | 19.51 | <0.0001 |
| Hampel | 1.49 | 19.74 | <0.0001 |
| Expression abundance |  |  |  |
| Huber | -0.60 | -178.62 | <0.0001 |
| bisquare | -0.60 | -179.78 | <0.0001 |
| Hampel | -0.59 | -179.64 | <0.0001 |
| *Gene body methylation^1^* |  |  |  |
| *Huber* | *-0.32* | *-5.25* | *<0.0001* |
| *bisquare* | *-0.32* | *-5.25* | *<0.0001* |
| *Hampel* | *-0.30* | *-5.00* | *<0.0001* |
| Promoter methylation |  |  |  |
| Huber | *0.20* | *4.93* | <0.0001 |
| bisquare | *0.20* | *4.81* | <0.0001 |
| Hampel | *0.20* | *4.98* | <0.0001 |
| log(Gene length) *^1^* |  |  |  |
| Huber | 0.001 | 0.18 | 0.8544 |
| bisquare | 0.001 | 0.12 | 0.9008 |
| Hampel | 0.001 | 0.11 | 0.9150 |
| Blood |  |  |  |
| Intercept |  |  |  |
| Huber | 1.90 | 28.23 | <0.0001 |
| bisquare | 1.90 | 28.34 | <0.0001 |
| Hampel | 1.90 | 28.65 | <0.0001 |
| Expression abundance |  |  |  |
| Huber | 0.55 | -232.00 | <0.0001 |
| bisquare | 0.55 | -233.63 | <0.0001 |
| Hampel | 0.55 | -235.64 | <0.0001 |
| *Gene body methylation^1^* |  |  |  |
| *Huber* | *-0.37* | *-6.41* | *<0.0001* |
| *bisquare* | *-0.36* | *-6.40* | *<0.0001* |
| *Hampel* | *-0.37* | *-6.67* | *<0.0001* |
| Promoter methylation |  |  |  |
| Huber | 0.27 | *6.73* | <0.0001 |
| bisquare | 0.27 | *6.76* | <0.0001 |
| Hampel | 0.29 | *7.33* | <0.0001 |
| log(Gene length) *^1^* |  |  |  |
| Huber | -0.04 | -4.77 | <0.0001 |
| bisquare | -0.04 | -4.78 | <0.0001 |
| Hampel | -0.04 | -6.67 | <0.0001 |

Robust Regression Analyses for the model used in Table 2.

| **Predictors** | **Estimate of *β*** | **t-value** | **Significance** |
| --- | --- | --- | --- |
| Brain |  |  |  |
| Intercept |  |  |  |
| Quantile | 1.53 | 21.20 | < 0.0001 |
| Huber | 1.50 | 19.48 | < 0.0001 |
| bisquare | 1.51 | 19.69 | < 0.0001 |
| Hampel | 1.49 | 19.59 | < 0.0001 |
| Expression abundance |  |  |  |
| Quantile | -0.61 | -190.89 | < 0.0001 |
| Huber | -0.60 | -178.81 | < 0.0001 |
| bisquare | -0.59 | -179.92 | < 0.0001 |
| Hampel | -0.59 | -179.78 | < 0.0001 |
| *Gene body methylation^1^* |  |  |  |
| *Quantile* | *-0.19* | *-3.30* | *0.0010* |
| *Huber* | *-0.23* | *-3.70* | *0.0002* |
| *bisquare* | *-0.23* | *-3.74* | *0.0002* |
| *Hampel* | *-0.21* | *-3.46* | *0.0005* |
| *TE methylation* |  |  |  |
| *Quantile* | *-0.16* | *-4.86* | *< 0.0001* |
| *Huber* | *-0.23* | *-5.52* | *< 0.0001* |
| *bisquare* | *-0.22* | *-5.43* | *< 0.0001* |
| *Hampel* | *-0.23* | *-5.64* | *< 0.0001* |
| Promoter methylation |  |  |  |
| Quantile | *0.12* | *3.59* | *0.0003* |
| Huber | *0.19* | *4.55* | *< 0.0001* |
| bisquare | *0.18* | *4.44* | *< 0.0001* |
| Hampel | *0.18* | *4.60* | *< 0.0001* |
| log(Gene length) *^1^* |  |  |  |
| Quantile | 0.01 | 1.18 | 0.24 |
| Huber | 0.02 | 1.61 | 0.11 |
| bisquare | 0.02 | 1.54 | 0.12 |
| Hampel | 0.01 | 1.54 | 0.12 |
| Blood |  |  |  |
| Intercept |  |  |  |
| Quantile | 1.84 | 27.93 | <0.0001 |
| Huber | 1.89 | 28.10 | <0.0001 |
| bisquare | 1.88 | 28.13 | <0.0001 |
| Hampel | 1.87 | 28.47 | <0.0001 |
| Expression abundance |  |  |  |
| Quantile | -0.55 | -218.70 | <0.0001 |
| Huber | -0.55 | -231.70 | <0.0001 |
| bisquare | -0.55 | -235.28 | <0.0001 |
| Hampel | -0.55 | -235.28 | <0.0001 |
| *Gene body methylation^1^* |  |  |  |
| *Quantile* | *-0.22* | *-3.50* | *0.0005* |
| *Huber* | *-0.30* | *-5.02* | *<0.0001* |
| *bisquare* | *-0.29* | *-5.01* | *<0.0001* |
| *Hampel* | *-0.29* | *-5.07* | *<0.0001* |
| *TE methylation* |  |  |  |
| *Quantile* | *-0.16* | *-3.24* | *0.0012* |
| *Huber* | *-0.19* | *-4.84* | *<0.0001* |
| *bisquare* | *-0.19* | *-4.70* | *<0.0001* |
| *Hampel* | *-0.21* | *-5.37* | *<0.0001* |
| Promoter methylation |  |  |  |
| Quantile | 0.20 | *5.09* | <0.0001 |
| Huber | 0.26 | *6.31* | <0.0001 |
| bisquare | 0.26 | *6.33* | <0.0001 |
| Hampel | 0.27 | *6.86* | <0.0001 |
| log(Gene length) *^1^* |  |  |  |
| Quantile | -0.03 | -3.24 | *0.0012* |
| Huber | -0.03 | -3.20 | *0.0014* |
| bisquare | -0.03 | -3.24 | *0.0012* |
| Hampel | -0.03 | -3.22 | *0.0013* |
