# Supplementary material for: DNA methylation and transcriptional noise
Source: Epigenetics Chromatin. 2013 Apr 26;6:9. doi: 10.1186/1756-8935-6-9 (PMC3641963; doi:10.1186/1756-8935-6-9)
Supplement: Additional file 6 — Multiple linear regression analyses incorporating individual factors indicate little effect of between-individual DNA methylation on transcriptional noise. [file 1756-8935-6-9-S6.doc]

**Additional File 6.** Multiple linear regression analyses incorporating individual factors indicate little effect of between-individual DNA methylation on transcriptional noise.

| **Predictors** | **Estimate of *β*** | **t-value** | **Significance** | **a** |
| --- | --- | --- | --- | --- |
| Brain |  |  |  |  |
| (Intercept) | 1.340 | 24.05 | < 10^-4^ |  |
| Intercept:ind2 | 0.0490 | 0.927 | 0.354 | 3.89 |
| Intercept:ind3 | 0.0240 | 0.46 | 0.646 |  |
| expression | -0.599 | -275.5 | < 10^-4^ | 1.03 |
| GBMT | -0.397 | -6.822 | < 10^-4^ | 2.00 |
| GBMT:ind2 | -0.0676 | -0.931 | 0.352 | 4.05 |
| GBMT:ind3 | -0.0213 | -0.3 | 0.764 |  |
| PMT | 0.230 | 10.79 | < 10^-4^ | 1.04 |
| log(gene length) | 0.0254 | 4.511 | < 10^-4^ | 1.30 |
| *Adj-R^2^* |  |  |  | 0.87 |
|  |  |  |  |  |

^a^Variation inflation factor approximated as (generalized VIF)^1/(2*d.f.)^.
