# Supplementary material for: DNA methylation and transcriptional noise
Source: Epigenetics Chromatin. 2013 Apr 26;6:9. doi: 10.1186/1756-8935-6-9 (PMC3641963; doi:10.1186/1756-8935-6-9)
Supplement: Additional file 7 — List of microarray datasets used in this study. [file 1756-8935-6-9-S7.doc]

Additional File 7. Sample information used in analysis after quality control. All samples in each tissue have correlation higher than 0.8 with each other.

| Tissue | Index | Experiment ID | sample ID | Version  (AFX-U133) | Gender | Age |
| --- | --- | --- | --- | --- | --- | --- |
| Brain | 1 | GSE3790 | GSM86929 | 1 | F | 37 |
| Brain | 2 | GSE3790 | GSM86931 | 1 | M | 22 |
| Brain | 3 | GSE3790 | GSM86933 | 1 | M | 37 |
| Brain | 4 | GSE3790 | GSM86937 | 1 | M | 72 |
| Brain | 5 | GSE3790 | GSM86938 | 1 | M | 81 |
| Brain | 6 | GSE3790 | GSM86939 | 1 | F | 68 |
| Brain | 7 | GSE3790 | GSM86940 | 1 | M | 29 |
| Brain | 8 | GSE3790 | GSM86942 | 1 | M | 67 |
| Brain | 9 | GSE3790 | GSM86943 | 1 | M | 22 |
| Brain | 10 | GSE3790 | GSM86947 | 1 | M | 51 |
| Brain | 11 | GSE3790 | GSM86948 | 1 | F | 68 |
| Brain | 12 | GSE3790 | GSM86951 | 1 | F | 61 |
| Blood | 1 | GSE3365 | GSM76115 | 1 | M | 60 |
| Blood | 2 | GSE3365 | GSM76116 | 1 | M | 53 |
| Blood | 3 | GSE3365 | GSM76117 | 1 | M | 54 |
| Blood | 4 | GSE3365 | GSM76118 | 1 | M | 53 |
| Blood | 5 | GSE3365 | GSM76119 | 1 | M | 54 |
| Blood | 6 | GSE3365 | GSM76120 | 1 | F | 47 |
| Blood | 7 | GSE3365 | GSM76121 | 1 | F | 48 |
| Blood | 8 | GSE3365 | GSM76122 | 1 | F | 47 |
| Blood | 9 | GSE3365 | GSM76123 | 1 | F | 42 |
| Blood | 10 | GSE3365 | GSM76124 | 1 | M | 54 |
| Blood | 11 | GSE3365 | GSM76125 | 1 | F | 56 |
| Blood | 12 | GSE3365 | GSM76126 | 1 | F | 52 |
| Blood | 13 | GSE3365 | GSM76127 | 1 | F | 45 |
| Blood | 14 | GSE3365 | GSM76128 | 1 | F | 51 |
| Blood | 15 | GSE3365 | GSM76129 | 1 | M | 57 |
| Blood | 16 | GSE3365 | GSM76130 | 1 | M | 45 |
| Blood | 17 | GSE3365 | GSM76131 | 1 | M | 36 |
| Blood | 18 | GSE3365 | GSM76132 | 1 | M | 42 |
| Blood | 19 | GSE3365 | GSM76133 | 1 | F | 35 |
| Blood | 20 | GSE3365 | GSM76134 | 1 | M | 31 |
| Blood | 21 | GSE3365 | GSM76135 | 1 | F | 31 |
| Blood | 22 | GSE3365 | GSM76136 | 1 | F | 42 |
| Blood | 23 | GSE3365 | GSM76137 | 1 | F | 54 |
| Blood | 24 | GSE3365 | GSM76138 | 1 | F | 47 |
| Blood | 25 | GSE3365 | GSM76139 | 1 | M | 49 |
| Blood | 26 | GSE3365 | GSM76141 | 1 | F | 28 |
| Blood | 27 | GSE3365 | GSM76142 | 1 | F | 39 |
| Blood | 28 | GSE3365 | GSM76143 | 1 | M | 31 |
| Blood | 29 | GSE3365 | GSM76144 | 1 | M | 42 |
| Blood | 30 | GSE3365 | GSM76145 | 1 | F | 44 |
| Blood | 31 | GSE3365 | GSM76146 | 1 | M | 42 |
| Blood | 32 | GSE3365 | GSM76147 | 1 | M | 33 |
| Blood | 33 | GSE3365 | GSM76148 | 1 | M | 40 |
| Blood | 34 | GSE3365 | GSM76149 | 1 | M | 42 |
| Blood | 35 | GSE3365 | GSM76150 | 1 | M | 50 |
| Blood | 36 | GSE3365 | GSM76151 | 1 | M | 47 |
| Blood | 37 | GSE3365 | GSM76153 | 1 | M | 40 |
| Blood | 38 | GSE3365 | GSM76154 | 1 | M | 27 |
| Blood | 39 | GSE3365 | GSM76155 | 1 | M | 25 |
| Blood | 40 | GSE3365 | GSM76156 | 1 | M | 40 |
